# Association of age, sex and race with prescription of anti-osteoporosis medications following low-energy hip fracture in a retrospective registry cohort

**DOI:** 10.1371/journal.pone.0278368

**Published:** 2022-12-01

**Authors:** Graeme Hoit, Daniel B. Whelan, Amit Atrey, Bheeshma Ravi, Gareth Ryan, Earl Bogoch, Aileen M. Davis, Amir Khoshbin

**Affiliations:** 1 Division of Orthopaedic Surgery, Department of Surgery, University of Toronto, Toronto, Ontario, Canada; 2 Institute of Health Policy, Management and Evaluation, Dalla Lana School of Public Health, University of Toronto, Toronto, Ontario, Canada; 3 Division of Orthopaedic Surgery, Unity Health – St. Michael’s Hospital, Toronto, Ontario, Canada; 4 Division of Orthopaedic Surgery, Sunnybrook Health Sciences Centre, Toronto, Ontario, Canada; 5 Faculty of Medicine, University of Toronto, Toronto, Ontario, Canada; Medical College of Wisconsin, UNITED STATES

## Abstract

**Background:**

Initiation of anti-osteoporosis medications after hip fracture lowers the risk of subsequent fragility fractures. Historical biases of targeting secondary fracture prevention towards certain groups may result in treatment disparities. We examined associations of patient age, sex and race with anti-osteoporosis medication prescription following hip fracture.

**Methods:**

A cohort of patients with a hip fracture between 2016–2018 was assembled from the American College of Surgeons National Surgical Quality Improvement Program registry. Patients on anti-osteoporosis medications prior to admission were excluded. Multivariable logistic regression was used to determine adjusted associations between patient age, sex and race and their interactions with prescription of anti-osteoporosis medications within 30 days of surgery.

**Results:**

In total, 12,249 patients with a hip fracture were identified with a median age of 82 years (IQR: 73–87), and 67% were female (n = 8,218). Thirty days postoperatively, 26% (n = 3146) of patients had been prescribed anti-osteoporosis medication. A significant interaction between age and sex with medication prescription was observed (p = 0.04). Male patients in their 50s (OR:0.75, 95%CI:0.60–0.92), 60s (OR:0.81, 95%CI:0.70–0.94) and 70s (OR:0.89, 95%CI:0.81–0.97) were less likely to be prescribed anti-osteoporosis medication compared to female patients of the same age. Patients who belonged to minority racial groups were not less likely to receive anti-osteoporosis medications than patients of white race.

**Interpretation:**

Only 26% of patients were prescribed anti-osteoporosis medications following hip fracture, despite consensus guidelines urging early initiation of secondary prevention treatments. Given that prescription varied by age and sex, strategies to prevent disparities in secondary fracture prevention are warranted.

## Introduction

Osteoporotic or low-energy hip fractures are common and are associated with high morbidity, mortality and healthcare expenditures [1–3]. Patients who suffer a low-energy hip fracture are at imminent risk of future fragility fractures, including a second hip fracture [4, 5]. Ten percent of patients hospitalized for a hip fracture will suffer a second hip fracture [4, 5], with the highest risk of subsequent fracture within the first year [6–8]. There is strong evidence that initiation of anti-osteoporosis medication following hip fractures can significantly lower the risk of subsequent fractures [9] and may be associated with overall reduced mortality [10]. Accordingly, guidelines from Canada [11], the United States [12], the United Kingdom [13] and international multi-stakeholder coalitions [14] have recommended the prescription of anti-osteoporosis medications following low-energy hip fracture for secondary prevention.

Despite the proven benefits of anti-osteoporosis medications in these patients, several studies have demonstrated a failure of healthcare providers to prescribe these medications following hospitalization for hip fracture [15–18]. In fact, a Public Health Agency of Canada report on the osteoporosis care gap found that the percentage of patients prescribed anti-osteoporosis medications following fracture decreased in the 2010s compared to previous decades [19]. Accordingly, current guidelines have encouraged the use of systemic screening and treatment protocols, such as Fracture Liaison Services, in order to minimize the effect of unconscious biases that could inhibit initiation of anti-osteoporotic medications in certain groups [20–22]. This strategy was, in part, likely motivated by several historical studies documenting the failure of practitioners to recognize or treat osteoporosis in male patients [23–25].

With the current consensus guidelines and use of standardized treatment protocols, understanding which patient characteristics continue to be associated with prescription of anti-osteoporosis medications following hip fracture could help identify those who remain at risk of being missed for secondary prevention. The objective of this study was to determine whether patient sex, age and race are associated with anti-osteoporosis medication prescription following low-energy hip fracture.

## Materials and methods

### Study design, data source and research ethics

We conducted a registry-based cohort study using the American College of Surgeons National Surgical Quality Improvement Program (ACS-NSQIP) registry. ACS-NSQIP is a prospectively collected and audited registry of patients who are followed for thirty days after undergoing common surgical procedures in 722 North American hospitals, of which 96 are in Canada. [26, 27] Audited coders review inpatient and outpatient records to track the occurrence of peri-operative and post-operative complications and outcomes of interest within thirty days of surgery. The accuracy and reproducibility of ACS-NSQIP coding has been previously studied in several surgical sub-specialities including orthopaedics. [28–36]

We report our study according to the Strengthening of the Reporting of Observational Studies in Epidemiology (STROBE) guidelines and the PROGRESS Framework (place of residence, race, occupation, gender, religion, education, socioeconomic status, or social capital) for identification of health inequity [37]. The study protocol was approved by the Research Ethics Board at St. Michael’s Hospital–Unity Health, Toronto, Ontario, Canada. The need for individual patient consent was waived by the ethics committee.

### Patients

We identified all patients in the ACS-NSQIP registry over 50 years old [11] who underwent surgery for a low-energy hip fracture between January 1, 2016, and December 31, 2018, using the ACS-NSQIP hip fracture targeted procedure variables and cross-referenced with Current Procedural Terminology (CPT) billing codes (S1 Table). The beginning of patient accrual in 2016 was chosen to coincide with the introduction of hip fracture-specific variables into the ACS-NSQIP registry as part of their targeted procedure initiative [26]. We excluded patients with pathologic fractures, disseminated cancer, active infections, those with chronic kidney disease [38], those who died within 30-days of their hospitalization, those with missing or unknown race and those who were previously prescribed anti-osteoporosis medication prior to their hip fracture (Table 1). Patients with multi-system injuries or high energy injuries are excluded from the ACS-NSQIP registry [26] and thus excluded from our study.

**Table 1:** Cohort assembly.

Inclusion and Exclusion Criteria	No.
Inclusion criteria	
Patients ≥50yr with surgically treated hip fractures at an ACS-NSQIP institution during study period	28,421
Exclusion criteria	
Pathologic Fracture	1,173
Disseminated Cancer	419
Current Infection	1,082
History of Chronic Kidney Disease or Dialysis	349
Died within 30 days of surgery	1,152
Treatment with anti-osteoporosis medication prior to fracture	7,589
Race status not reported or unknown	4,408
Eligible patients (total study cohort size)	12,249

Abbreviations: ACS-NSQIP: American College of Surgeons National Surgical Quality Improvement Program registry; yr: years.

### Main variables of interest

The main variables of interest were sex, age and race. Patient sex is coded in the ACS-NSQIP registry with the choices of “Male” and “Female”. Patient gender is not captured in the registry, and thus is not analyzed in this study. Within ACS-NSQIP, age is truncated at 90 years. Accordingly, age was analyzed as a continuous variable of whole decades (i.e., 50s, 60s, 70s, 80s, 90s). In ACS-NSQIP, race can be either self-assigned or assigned by institutional personnel as per internal practices and is coded as one of: i) American Indian or Alaska Native, ii) Asian, iii) Black or African American, iv) Native Hawaiian or Pacific Islander, or v) White. Hispanic ethnicity is reported as a separate variable, which we included as a race category [26, 39].

### Covariates

Covariates included additional patient characteristics previously shown to explain outcomes following hip fracture surgery, including body mass index (BMI) [40], Anesthesia Society of America class [41] and medical comorbidity [42]. Medical comorbidity was operationalized using the previously validated modified frailty index (mFI-5)—a composite score consisting of five variables captured within ACS-NSQIP used to predict post-operative mortality [43, 44]. The variables in the mFI-5 score are: patient functional status, diabetes, history of chronic obstructive pulmonary disorder (COPD), history of congestive heart failure (CHF) and hypertension requiring medication.

Covariates also included injury and treatment factors previously shown to affect clinical outcomes in patients with hip fractures, including location of fracture [42], type of surgical treatment, hospital length of stay [45], a hospital-level implementation of a standardized hip fracture program [46] and medical co-management of patient care [47]. We divided the type of surgical treatment into two categories: those who underwent fixation thus requiring bone healing (screw constructs, plate and screw constructs and cephalomedullary nailing) and those who underwent replacement surgery (hemi- or total hip arthroplasty). ACS-NSQIP defines an institutional standardized hip fracture program as “including standard order sets, standard clinical pathways and standard protocols designed to improve outcomes” in patients with hip fractures [26]. ACS-NSQIP defines medical co-management as the documented involvement of medical subspecialty physicians (internal medicine, geriatrics) in patient clinical care including pre-operative consultation and postoperative visits [26].

### Anti-osteoporosis medication ascertainment: Previous use and post-fracture use (primary outcome)

Previous anti-osteoporosis medication (or “bone protective medication”) prescription is a binary variable within the ACS-NSQIP registry (yes/no). This includes prescribed oral/intranasal medications (bisphosphonates, raloxifene, vitamin D and calcitonin) taken for at least two weeks and within 30 days prior to admission, and intravenous or injected medication (bisphosphonates, teriparatide, denosumab, calcitonin) prescribed within 1 year [26]. Other medications prescribed for the purpose of bone protection (i.e. hormone replacement therapy, therapeutic testosterone) can be considered for this variable on a case-by-case basis and are adjudicated by the treating surgeon or hospital ACS-NSQIP surgeon champion [26]. Specification of medication type, dosage and duration is not captured by ACS-NSQIP. Herbal remedies, non-prescription multivitamins or calcium preparations are not coded as anti-osteoporosis medication in ACS-NSQIP [48, 49].

The primary outcome was an ACS-NSQIP recorded prescription for anti-osteoporosis medication within 30 days of hip fracture surgery, which includes both prescriptions initiated during inpatient stay and those issued after hospital discharge by outside providers in the community or at secondary inpatient institutions. Post-surgery anti-osteoporosis medications include the same aforementioned medications as those documented for previous use. Prescription is recorded as a binary variable (yes, no) without further specification of medication type, dose or duration [26].

### Statistical analysis

We performed multivariable logistic regression to measure independent associations between our variables of interest (patient age, sex and race) and the prescription of anti-osteoporosis medication within thirty days of hip fracture surgery. Due to small proportions of patients within certain race categories, race was re-categorized as Asian, Black, Hispanic, Indigenous and White [39]. Indigenous race was inclusive of American Indians, Native Alaskans, Native Hawaiians and Pacific Islanders. We included additional exposure variables thought to be confounders based on existing literature [17, 20, 50] and clinical judgement. These included BMI, ASA score, mFI5 score, surgical fixation versus replacement, standardized hip fracture protocols, medical co-management and hospital length of stay [26]. Interaction terms between exposures of interest (age*sex, age*race, sex*race, age*sex*race) were entered into the model and retained for final analysis if associated p-values were <0.05. Odds ratios (ORs) and 95% confidence intervals (95%CIs) were generated.

Baseline cohort characteristics were reported as means with standard deviations (SDs), medians with interquartile ranges (IQRs) or proportions as appropriate. Missing data was less than 1% for included covariates and imputation methods were not used. All analyses were performed using SAS statistical software version 9.4 (SAS Institute Inc, Cary NC).

## Results

After exclusion of 16,172 hospitalized hip fracture patients (Table 1), a total of 12,249 patients met the inclusion criteria (Table 2). The median age was 82 years (IQR 73–87) and 67% (n = 8,218) were female. The majority of patients were classified as white race (n = 10,923, 90%). Fourty-four percent (n = 5,360) of patients were treated at a hospital with a standardized hip fracture program, and 95% (n = 11,643) were co-managed by a member of the medical team during their admission.

**Table 2:** Cohort characteristics.

	All Patients	Female	Male
**Total, n (%)**	**12,249 (100%)**	**8,218 (100%)**	**4,031 (100%)**
**Patient and Injury Characteristics**			
Age in years, median (IQR)	82 (73–87)	83 (74–88)	79 (69–86)
50–59, n (%)	664 (5%)	340 (4%)	324 (8%)
60–69, n (%)	1,690 (14%)	972 (12%)	718 (18%)
70–79, n (%)	2,910 (24%)	1,866 (23%)	1,044 (26%)
80–89, n (%)	5,071 (41%)	3,577 (44%)	1,494 (37%)
90+, n (%)	1,914 (16%)	1,463 (18%)	451 (11%)
Female Sex, n (%)	8,218 (67%)	8,218 (100%)	0 (0%)
BMI in kg/m^2^, mean (SD)	24 (8)	24 (8)	25 (7)
Race			
American Indian or Native Alaskan	47 (0.4%)	30 (0.4%)	17 (0.4%)
Asian	268 (2%)	197 (2%)	71 (2%)
Black or African American	510 (4%)	312 (4%)	198 (5%)
Hispanic	487 (4%)	308 (4%)	179 (4%)
Native Hawaiian or Pacific Islander	14 (0.1%)	11 (0.1%)	3 (0.1%)
White	10,923 (90%)	7,360 (88%)	3,563 (89%)
Functional status			
Independent	9,988 (82%)	6,627 (81%)	3,361 (83%)
Partially Dependent	1951 (16%)	1,370 (17%)	581 (14%)
Totally Dependent	258 (2%)	186 (2%)	72 (2%)
Unknown	52 (0.4%)	35 (0.4%)	17 (0.4%)
Hypertension	8,158 (67%)	5,508 (67%)	2,650 (66%)
Diabetes	2,288 (19%)	1,382 (17%)	906 (22%)
COPD History	1,374 (11%)	858 (10%)	516 (13%)
CHF History	371 (3%)	215 (3%)	156 (4%)
ASA Class			
1	77 (1%)	51 (1%)	26 (1%)
2	2,381 (19%)	1,744 (21%)	637 (16%)
3	7,898 (64%)	5,258 (64%)	2,640 (65%)
4	1,874 (15%)	1,151 (14%)	723 (18%)
5	9 (0.1%)	6 (0.1%)	3 (0.1%)
Unknown	10 (0.1%)	8 (0.1%)	2 (0.1%)
Fracture Location			
Femoral Neck	4,699 (38%)	3,086 (38%)	1,613 (40%)
Intertrochanteric	6,935 (57%)	4,709 (57%)	2,226 (55%)
Subtrochanteric	615 (5%)	423 (5%)	192 (5%)
**Treatment Characteristics**			
Surgical Method			
Replacement	4,437 (36%)	2,905 (35%)	1,532 (38%)
Fixation	7,812 (64%)	5,313 (65%)	2,499 (62%)
Standardized Hip Fracture Program	5,360 (44%)	3,605 (44%)	1,755 (44%)
Hip Fracture Co-Management	11,643 (95%)	7,824 (95%)	3,819 (95%)
Hospital length of stay, median days (IQR)	5 (4–7)	5 (4–6)	5 (4–7)

Percentages are listed as column percentages (i.e., percentage of total, percentage of females, percentage of males, respectively).

Abbreviations: IQR: interquartile range, BMI: body mass index, SD: standard deviation, COPD: chronic obstructive pulmonary disease, CHF: congestive heart failure, ASA: Anesthesia Society of America

Within thirty days of surgical treatment for hip fracture, 26% (n = 3,146) of patients had been prescribed anti-osteoporosis medication, including 26% (n = 2,159) of females and 24% (n = 987) of males. Within our multivariable regression analysis, a significant interaction was observed between age and sex (p = 0.039) resulting in retention in the model (Table 3 and S2 Table). Male patients in their 50s (OR:0.75, 95%CI:0.60–0.92), 60s (OR:0.81, 95%CI:0.70–0.94) and 70s (OR:0.89, 95%CI:0.81–0.97) had lower odds of being prescribed anti-osteoporosis medications compared to female patients of the same age. No association between patient sex and postoperative anti-osteoporosis medication was observed for patients older than age 80 (80s: OR 0.96, 95%CI:0.87–1.07; 90s: OR 1.05, 95%CI:0.98–1.12). Age was not independently associated with prescribing medication (p = 0.14) in the model outside of the interaction of age and sex. There were no differences in association with our primary outcome amongst those of Asian, Black or Hispanic race compared to those of White race. We observed an increased odds of anti-osteoporosis medication initiation in patients classified as Indigenous race, inclusive of American Indians, Native Alaskans, Native Hawaiians, Pacific Islanders (OR 2.01, 95%CI:1.19–3.38).

**Table 3:** Adjusted associations of exposures and covariates with prescription of anti-osteoporosis medication.

	Odds Ratio^a^ (95% CI)	P Value^a^
**Main Exposures**		
Age (Decade)	-	0.14
Male sex	-	0.010
Age* Sex	-	0.039
Male sex within patients age 50–59	0.75 (0.60–0.92)	0.007
Male sex within patients age 60–69	0.81 (0.70–0.94)	0.005
Male sex within patients age 70–79	0.89 (0.81–0.97)	0.011
Male sex within patients age 80–89	0.96 (0.87–1.07)	0.47
Male sex within patients age 90+	1.05 (0.90–1.23)	0.55
Age within patients of female sex	0.96 (0.92–1.01)	0.14
Age within patients of male sex	1.05 (0.98–1.12)	0.15
Race (Reference: White)	-	0.036
Asian	0.83 (0.62–1.11)	0.21
Black	0.87 (0.70–1.08)	0.21
Hispanic	0.94 (0.76–1.16)	0.58
Indigenous	2.01 (1.19–3.38)	0.009

Abbreviations: CI: confidence interval.

^a^Odds ratios, 95% CIs, and p-values produced using multivariable logistic regression adjusting for BMI, ASA class, mFI5 comorbidity score, fracture location, type of surgical treatment, hospital length of stay, whether or not they were treated at a hospital with a standardized hip fracture program and whether or not they were co-managed by a medical specialty physician. Odds ratios for all covariates are shown in S2 Table.

## Discussion

In this retrospective, registry-based study of North American post-surgical patients with low-energy hip fractures, we found that male patients younger than 80 years old were less likely to be prescribed anti-osteoporosis medications post-operatively compared to similarly aged female patients. Only one-quarter of patients were prescribed anti-osteoporosis medications within thirty days of surgery, despite consensus guidelines recommending for secondary prevention for all patients in this population [11–14]. This evidence suggests that secondary fracture prevention following low-energy hip fracture continues to be sub-optimal, and that treatment disparities between sexes persist despite the recent widespread implementation of standardized hip fracture programs and fragility fracture screening programs designed to optimize post-fracture care in these patients [20–22].

The proportion of patients who were prescribed anti-osteoporosis medication—26%—while low, actually over-estimates the number of patients truly prescribed effective secondary fracture prevention medication [15–18]. In a 2020 report, the Public Health Agency of Canada (PHAC) estimated 25% of hip fracture patients were started on anti-osteoporosis medication within one year of fracture [19], an estimate consistent with our findings. However, unlike the ACS-NSQIP registry, the PHAC study did not consider vitamin D as an anti-osteoporosis medication, given evidence that vitamin D supplementation alone does not reduce risk of secondary osteoporotic fractures [51]. Thus, it is likely that patients who met our inclusion criteria at ACS-NSQIP participating hospitals were prescribed effective treatment at an even lower rate than those studied by the PHAC in 2020.

Most previous studies linking male sex with inequities in secondary fracture prevention did so prior to development of consensus guidelines and widespread implementation of standardized care plans for hip fracture patients [23–25]. These guidelines have since made it clear that the initiation of anti-osteoporosis medications is imperative for preventing further fragility fractures. Our study demonstrates that despite standardized hip fracture programs at many treating hospitals (44%) and the frequent co-management of patients by non-surgical medical teams (94%), the majority of patients (74%) were not prescribed anti-osteoporosis medications following hip fracture surgery, and male patients had lower odds of receiving prescriptions. These findings are consistent with those in an analysis of post-fragility fracture care using the Australian and New Zealand Hip Fracture Registry, where despite widespread implementation of Orthogeriatric co-management programmes during hospitalization, prescription of secondary prevention medication was inconsistent and remained low [52]. These findings and combined with those in our study suggest that in-patient optimization of hip fracture care alone may not be sufficient to ensure adequate post-admission treatment of osteoporosis.

Previous studies have examined societal perceptions of osteoporosis and demonstrated that it is viewed predominantly as a ‘woman’s disease’ [53–55]. Existence of this belief among healthcare providers could offer explanation for the sex-based prescribing differences found in our study. While the overall difference in medication prescription between male and female patients was only 2%, the largest disparity in medication prescription between sexes was demonstrated in the younger patients within our cohort. Among patients aged 50–59, male sex was associated with a 25% decrease in the odds of being prescribed anti-osteoporosis medications. This association progressively decreased in magnitude with each decade of life, with no differences in treatment between sexes in those aged 80 or older. To our knowledge, this study is the first to examine the influence of patient age on sex disparities in osteoporosis care.

Our findings suggest a role for structured screening and treatment programs to avoid disparities in secondary prevention of fragility fractures between sexes and patients of varying ages. Recently, evidence presented from the Ontario Osteoporosis Strategy program suggests that standardized screening facilitates medication prescription and can eliminate sex- and age-based health inequities in secondary fracture prevention [20, 56]. Ansari *et al*. [20] demonstrated similar rates of treatment initiation between sexes with 66% of high-risk men and 68% of high-risk women (68%) started on anti-osteoporosis medications within six months of initial fracture–a prescription rate much higher than what we found (26%). One possible explanation for this discrepancy beyond the structured screening program is our relatively short duration of post-operative follow-up. It is possible that a proportion of patients who were not prescribed anti-osteoporosis medications within thirty days postoperatively had them prescribed by a provider at a later date. However, the prompt identification and treatment of patients at risk of future fractures is of critical importance, in particular due to the imminent risk repeat fracture within one-year [4, 5]. Because prior history of hip fracture is not one of the medical history items captured by ACS-NSQIP, a proportion of patients identified in our study may have indeed been those with repeat fractures who were not given anti-osteoporosis medication at initial fracture. The addition of previous hip fracture as a medical history datapoint in the ACS-NSQIP registry would provide benefit for future research on secondary fracture incidence and prevention. Additionally, our prescription rate of 26% exceeds that of many registry or population level studies with longer follow-up durations and exclusion of vitamin D due to its questionable efficacy at fracture risk reduction [15–18]. The clinical implication of these findings is that strategies aimed at increasing anti-osteoporosis medication prescription for males, particularly young males are warranted, including system-based solutions like automatic orders that require override to negate.

Previous epidemiology-based research has demonstrated racial and ethnic differences in predisposition to fragility fractures [57] and racial disparities in treatment and outcomes of osteoporosis [58–61]. Unlike these studies, we found no differences between patients of White race and those classified as Asian, Black or African American, or Hispanic in the prescription of anti-osteoporosis medication after hip fracture. However, those classified as Indigenous were more likely to be prescribed medication. The sample of indigenous patients within our study was very small (n = 61), and thus this estimate is prone to error. In contrast to our finding, prior research by Leslie et al has demonstrated a post-fracture treatment disparity in the indigenous population [61], despite a known higher incidence of osteoporosis and fractures within indigenous groups. [62–64] Optimistically, it is possible that this research has since increased the likelihood of indigenous patients being treated with anti-osteoporosis medications, though it is more likely that characteristic differences between the population studied by Leslie et al and those who presented for treatment to the hospitals included in our study account for this discrepancy [61]. Further research with a larger sample is required to explore this finding.

### Limitations

This study has several limitations. First, as with all registry studies, our results are subject to limitations in ACS-NSQIP data inclusions and the coding of the variables and exposures of interest. As such, we cannot comment on other potentially important factors often used for decision- making in osteoporosis care, such as vitamin D levels, bone density testing results or contraindications to medications other than chronic kidney disease, such as allergies. However, consensus guidelines consider a low-energy hip fracture in those over 50 years old sufficient to make the diagnosis of osteoporosis, and accordingly recommend initiation of secondary prevention medications [11–14]. Within the registry, sex is limited to male versus female coding and does not allow for reporting of intersex individuals, nor does it report variables related to gender. Sex differences between males and females reflect the biological and physiological differences. In osteoporosis care, it is well understood that sex-specific physiology results in a greater need for osteoporosis care amongst females due to increase prevalence [65]. However, gender-based differences in the socially driven roles and perception of disease and treatment likely also influence secondary prevention prescription for fragility fractures [65]. While sex and gender often do coincide, they are discrete entities, [66] and as such, our study can only comment on sex-based differences in the prescription of anti-osteoporosis medication. Future research evaluating the influence of gender on secondary prevention prescription is needed. Additionally, a large number of hospitalized hip fracture patients were excluded (n = 4,408) from our study, as a result of having “unknown or not reported” race. Within the ACS-NSQIP registry, race categories are limited to those offered in the US Census Bureau, and do not include some prominent minority groups, such as South Asian or Middle Eastern, and also do not allow for reporting mixed-race individuals [26]. In ACS-NSQIP, having “unknown or not reported” race is due to the patient’s race not being recorded in the medical record, either by not self-reporting or in not being assigned by the institution. Thus, it is possible that many of those recorded as “unknown or not reported” race identify with a racial group not represented by the available categories, which could limit the generalizability of our findings within certain groups. We conducted a post-hoc sensitivity analysis including those patients with unknown race as a separate racial category (S3 Table), which did not meaningfully change the conclusions of our study. Lastly, for the purpose of this study, we chose to analyze those of Hispanic ethnicity as a distinct race category. Previous sociologic research demonstrates that the majority of those identifying as of Hispanic ethnicity in the United States also view themselves as of Hispanic/Latino race despite this not being a race category within the US Census Bureau [67–69]. However, a minority of these individuals may identify as a separate racial category, such as White [70]. Based on limitations of our retrospective registry design and the available data, we could not honor these individual preferences for patients with coded Hispanic ethnicity status. Given that a goal of our study was to determine if osteoporosis care inequities were faced by those of minority groups, and previous research has demonstrated those identifying as ethnically Hispanic in the United States are racialized and often have a unique lived experience compared to those considered “White Americans” [67, 70–73], we felt it most appropriate to analyze all those reporting Hispanic ethnicity as of a distinct racial group.

## Conclusions

Only 26% of patients were prescribed anti-osteoporosis medications following hip fracture, despite consensus guidelines urging early initiation of secondary prevention treatments in this population. Given that prescription varied by age and sex, strategies need to be implemented to prevent sex- and age-based disparities in secondary fracture prevention.

## Supporting information

S1 Table Common Procedural Terminology (CPT) codes used for cohort building. (DOCX)

S2 Table Adjusted associations of exposures and covariates with prescription of anti-osteoporosis medication inclusive of all covariates. (DOCX)

S3 Table Sensitivity analysis: Adjusted associations of exposures and covariates with prescription of anti-osteoporosis medication inclusive of those with unknown race. (DOCX)

S1 File Anonymized final dataset for analysis (attached separately). (SAS7BDAT)

## References

[1] Gdalevich M, Cohen D, Yosef D, Tauber C. Morbidity and mortality after hip fracture: the impact of operative delay. Arch Orthop Trauma Surg. 2004;124(5):334–40. Epub 2004/04/20. doi: 10.1007/s00402-004-0662-9 . 15095097

[2] Endo Y, Aharonoff GB, Zuckerman JD, Egol KA, Koval KJ. Gender differences in patients with hip fracture: a greater risk of morbidity and mortality in men. J Orthop Trauma. 2005;19(1):29–35. Epub 2005/01/26. doi: 10.1097/00005131-200501000-00006 . 15668581

[3] Cooper C, Campion G, Melton LJ 3rd. Hip fractures in the elderly: a world-wide projection. Osteoporos Int. 1992;2(6):285–9. Epub 1992/11/01. doi: 10.1007/BF01623184 . 1421796

[4] Klotzbuecher CM, Ross PD, Landsman PB, Abbott TA 3rd, Berger M. Patients with prior fractures have an increased risk of future fractures: a summary of the literature and statistical synthesis. J Bone Miner Res. 2000;15(4):721–39. Epub 2000/04/26. doi: 10.1359/jbmr.2000.15.4.721 . 10780864

[5] Center JR, Bliuc D, Nguyen TV, Eisman JA. Risk of subsequent fracture after low-trauma fracture in men and women. JAMA. 2007;297(4):387–94. Epub 2007/01/25. doi: 10.1001/jama.297.4.387 . 17244835

[6] Nolan P, Tiedt L, Ellanti P, McCarthy T, Hogan N. Incidence of Non-Simultaneous Contralateral Second Hip Fractures: A Single-Center Irish Study. Cureus. 2020;12(10):e11154. Epub 2020/11/03. doi: 10.7759/cureus.11154 . 33133797 PMC7586354

[7] Fukushima T, Sudo A, Uchida A. Bilateral hip fractures. J Orthop Sci. 2006;11(5):435–8. Epub 2006/10/03. doi: 10.1007/s00776-006-1056-3 . 17013728

[8] Chen FP, Shyu YC, Fu TS, Sun CC, Chao AS, Tsai TL, et al. Secular trends in incidence and recurrence rates of hip fracture: a nationwide population-based study. Osteoporos Int. 2017;28(3):811–8. Epub 2016/11/11. doi: 10.1007/s00198-016-3820-3 . 27832325 PMC5306161

[9] Harvey NC, McCloskey EV, Mitchell PJ, Dawson-Hughes B, Pierroz DD, Reginster JY, et al. Mind the (treatment) gap: a global perspective on current and future strategies for prevention of fragility fractures. Osteoporos Int. 2017;28(5):1507–29. Epub 2017/02/09. doi: 10.1007/s00198-016-3894-y . 28175979 PMC5392413

[10] Bolland MJ, Grey AB, Gamble GD, Reid IR. Effect of osteoporosis treatment on mortality: a meta-analysis. J Clin Endocrinol Metab. 2010;95(3):1174–81. Epub 2010/01/19. doi: 10.1210/jc.2009-0852 . 20080842

[11] Papaioannou A, Morin S, Cheung AM, Atkinson S, Brown JP, Feldman S, et al. 2010 clinical practice guidelines for the diagnosis and management of osteoporosis in Canada: summary. CMAJ. 2010;182(17):1864–73. Epub 2010/10/14. doi: 10.1503/cmaj.100771 . 20940232 PMC2988535

[12] The Lancet Diabetes E. Osteoporosis: a roadmap to close the treatment gap. Lancet Diabetes Endocrinol. 2018;6(11):833. Epub 2018/10/17. doi: 10.1016/S2213-8587(18)30292-4 . 30322720

[13] Compston J, Cooper A, Cooper C, Gittoes N, Gregson C, Harvey N, et al. UK clinical guideline for the prevention and treatment of osteoporosis. Arch Osteoporos. 2017;12(1):43. Epub 2017/04/21. doi: 10.1007/s11657-017-0324-5 . 28425085 PMC5397452

[14] Conley RB, Adib G, Adler RA, Akesson KE, Alexander IM, Amenta KC, et al. Secondary Fracture Prevention: Consensus Clinical Recommendations from a Multistakeholder Coalition. J Bone Miner Res. 2020;35(1):36–52. Epub 2019/09/21. doi: 10.1002/jbmr.3877 . 31538675

[15] Kim SC, Kim MS, Sanfelix-Gimeno G, Song HJ, Liu J, Hurtado I, et al. Use of osteoporosis medications after hospitalization for hip fracture: a cross-national study. Am J Med. 2015;128(5):519–26 e1. Epub 2015/02/11. doi: 10.1016/j.amjmed.2015.01.014 . 25660252 PMC4414898

[16] Gonnelli S, Caffarelli C, Iolascon G, Bertoldo F, Letizia Mauro G, Patti A, et al. Prescription of anti-osteoporosis medications after hospitalization for hip fracture: a multicentre Italian survey. Aging Clin Exp Res. 2017;29(5):1031–7. Epub 2016/12/13. doi: 10.1007/s40520-016-0681-8 . 27943127

[17] Desai RJ, Mahesri M, Abdia Y, Barberio J, Tong A, Zhang D, et al. Association of Osteoporosis Medication Use After Hip Fracture With Prevention of Subsequent Nonvertebral Fractures: An Instrumental Variable Analysis. JAMA Netw Open. 2018;1(3):e180826. Epub 2019/01/16. doi: 10.1001/jamanetworkopen.2018.0826 . 30646034 PMC6324295

[18] Gamboa A, Duaso E, Marimon P, Sandiumenge M, Escalante E, Lumbreras C, et al. Oral bisphosphonate prescription and non-adherence at 12 months in patients with hip fractures treated in an acute geriatric unit. Osteoporos Int. 2018;29(10):2309–14. Epub 2018/08/05. doi: 10.1007/s00198-018-4622-6 . 30076454

[19] System CCDS. Osteoporosis and Related Fractures in Canada. Feb 2021;(200452).

[20] Ansari H, Beaton DE, Sujic R, Rotondi NK, Cullen JD, Slater M, et al. Equal treatment: no evidence of gender inequity in osteoporosis management in a coordinator-based fragility fracture screening program. Osteoporos Int. 2017;28(12):3401–6. Epub 2017/09/12. doi: 10.1007/s00198-017-4206-x . 28891035

[21] Giangregorio L, Papaioannou A, Cranney A, Zytaruk N, Adachi JD. Fragility fractures and the osteoporosis care gap: an international phenomenon. Semin Arthritis Rheum. 2006;35(5):293–305. Epub 2006/04/18. doi: 10.1016/j.semarthrit.2005.11.001 . 16616152

[22] Brown JP, Josse RG, Scientific Advisory Council of the Osteoporosis Society of C. 2002 clinical practice guidelines for the diagnosis and management of osteoporosis in Canada. CMAJ. 2002;167(10 Suppl):S1–34. Epub 2002/11/13. . 12427685 PMC134653

[23] Panneman MJ, Lips P, Sen SS, Herings RM. Undertreatment with anti-osteoporotic drugs after hospitalization for fracture. Osteoporos Int. 2004;15(2):120–4. Epub 2003/11/18. doi: 10.1007/s00198-003-1544-7 . 14618302

[24] Leslie WD, Giangregorio LM, Yogendran M, Azimaee M, Morin S, Metge C, et al. A population-based analysis of the post-fracture care gap 1996–2008: the situation is not improving. Osteoporos Int. 2012;23(5):1623–9. Epub 2011/04/09. doi: 10.1007/s00198-011-1630-1 . 21476038

[25] Kiebzak GM, Beinart GA, Perser K, Ambrose CG, Siff SJ, Heggeness MH. Undertreatment of osteoporosis in men with hip fracture. Arch Intern Med. 2002;162(19):2217–22. Epub 2002/10/24. doi: 10.1001/archinte.162.19.2217 . 12390065

[26] American College of Surgeons—National Surgical Quality Improvement Program—User Guide for the 2016 ACS NSQIP Procedure Targeted Participant USE Data File [Internet]. The ACS National Surgical Quality Improvement Program. 2015. https://www.facs.org/~/media/files/qualityprograms/nsqip/pt_nsqip_puf_userguide_2016.ashx.

[27] ACS-NSQIP. American College of Surgeons National Surgical Quality Improvement Program. 2017.

[28] Chughtai M, Gwam CU, Khlopas A, Newman JM, Curtis GL, Torres PA, et al. The Incidence of Postoperative Pneumonia in Various Surgical Subspecialties: A Dual Database Analysis. Surg Technol Int. 2017;30:45–51. Epub 2017/07/12. . 28695972

[29] Sodhi N, Piuzzi NS, Dalton SE, George J, Ng M, Khlopas A, et al. What Influence Does the Time of Year Have on Postoperative Complications Following Total Knee Arthroplasty? J Arthroplasty. 2018;33(6):1908–13. Epub 2018/01/21. doi: 10.1016/j.arth.2017.12.020 . 29352687

[30] Bedard NA, Pugely AJ, McHugh M, Lux N, Otero JE, Bozic KJ, et al. Analysis of Outcomes After TKA: Do All Databases Produce Similar Findings? Clinical orthopaedics and related research. 2018;476(1):52–63. Epub 2018/03/13. doi: 10.1007/s11999.0000000000000011 . 29529616 PMC5919249

[31] Bedard NA, Pugely AJ, McHugh MA, Lux NR, Bozic KJ, Callaghan JJ. Big Data and Total Hip Arthroplasty: How Do Large Databases Compare? J Arthroplasty. 2018;33(1):41–5 e3. Epub 2017/10/12. doi: 10.1016/j.arth.2017.09.003 . 29017802

[32] McIsaac DI, Hamilton GM, Abdulla K, Lavallee LT, Moloo H, Pysyk C, et al. Validation of new ICD-10-based patient safety indicators for identification of in-hospital complications in surgical patients: a study of diagnostic accuracy. BMJ Qual Saf. 2019. Epub 2019/08/24. doi: 10.1136/bmjqs-2018-008852 . 31439760

[33] Rolston JD, Han SJ, Chang EF. Systemic inaccuracies in the National Surgical Quality Improvement Program database: Implications for accuracy and validity for neurosurgery outcomes research. J Clin Neurosci. 2017;37:44–7. Epub 2016/11/20. doi: 10.1016/j.jocn.2016.10.045 . 27863971

[34] Neale JA, Reickert C, Swartz A, Reddy S, Abbas MA, Rubinfeld I. Accuracy of national surgery quality improvement program models in predicting postoperative morbidity in patients undergoing colectomy. Perm J. 2014;18(1):14–8. Epub 2014/03/15. doi: 10.7812/TPP/12-133 . 24626067 PMC3951025

[35] Awad MI, Shuman AG, Montero PH, Palmer FL, Shah JP, Patel SG. Accuracy of administrative and clinical registry data in reporting postoperative complications after surgery for oral cavity squamous cell carcinoma. Head Neck. 2015;37(6):851–61. Epub 2014/03/14. doi: 10.1002/hed.23682 . 24623622 PMC4976499

[36] Trickey AW, Wright JM, Donovan J, Reines HD, Dort JM, Prentice HA, et al. Interrater Reliability of Hospital Readmission Evaluations for Surgical Patients. Am J Med Qual. 2017;32(2):201–7. Epub 2016/02/26. doi: 10.1177/1062860615623854 . 26911664

[37] O’Neill J, Tabish H, Welch V, Petticrew M, Pottie K, Clarke M, et al. Applying an equity lens to interventions: using PROGRESS ensures consideration of socially stratifying factors to illuminate inequities in health. J Clin Epidemiol. 2014;67(1):56–64. Epub 2013/11/06. doi: 10.1016/j.jclinepi.2013.08.005 . 24189091

[38] Miller PD, Jamal SA, Evenepoel P, Eastell R, Boonen S. Renal safety in patients treated with bisphosphonates for osteoporosis: a review. J Bone Miner Res. 2013;28(10):2049–59. Epub 2013/08/03. doi: 10.1002/jbmr.2058 . 23907861

[39] National Research Council (US) Panel on Race EaHiLLAN, Bulatao RA, Cohen B, editors. 2, Racial and Ethnic Identification, Official Classifications, and Heatlh Disparities. Critical Perspectives on Racial and Ethnic Differences In Health in Late Life. Washington DC: National Academies Press; 2004. 20669464

[40] Akinleye SD, Garofolo G, Culbertson MD, Homel P, Erez O. The Role of BMI in Hip Fracture Surgery. Geriatr Orthop Surg Rehabil. 2018;9:2151458517747414. Epub 2018/02/23. doi: 10.1177/2151458517747414 . 29468090 PMC5813852

[41] Chen LH, Liang J, Chen MC, Wu CC, Cheng HS, Wang HH, et al. The relationship between preoperative American Society of Anesthesiologists Physical Status Classification scores and functional recovery following hip-fracture surgery. BMC Musculoskelet Disord. 2017;18(1):410. Epub 2017/10/12. doi: 10.1186/s12891-017-1768-x . 29017476 PMC5635509

[42] Yong EL, Ganesan G, Kramer MS, Howe TS, Koh JSB, Thu WP, et al. Risk Factors and Trends Associated With Mortality Among Adults With Hip Fracture in Singapore. JAMA Netw Open. 2020;3(2):e1919706. Epub 2020/02/15. doi: 10.1001/jamanetworkopen.2019.19706 . 32058551 PMC12124694

[43] Subramaniam S, Aalberg JJ, Soriano RP, Divino CM. New 5-Factor Modified Frailty Index Using American College of Surgeons NSQIP Data. J Am Coll Surg. 2018;226(2):173–81 e8. Epub 2017/11/21. doi: 10.1016/j.jamcollsurg.2017.11.005 . 29155268

[44] Traven SA, Reeves RA, Althoff AD, Slone HS, Walton ZJ. New Five-Factor Modified Frailty Index Predicts Morbidity and Mortality in Geriatric Hip Fractures. J Orthop Trauma. 2019;33(7):319–23. Epub 2019/02/08. doi: 10.1097/BOT.0000000000001455 . 30730361

[45] Nikkel LE, Kates SL, Schreck M, Maceroli M, Mahmood B, Elfar JC. Length of hospital stay after hip fracture and risk of early mortality after discharge in New York state: retrospective cohort study. BMJ. 2015;351:h6246. Epub 2015/12/15. doi: 10.1136/bmj.h6246 . 26655876 PMC4674667

[46] Arshi A, Rezzadeh K, Stavrakis AI, Bukata SV, Zeegen EN. Standardized Hospital-Based Care Programs Improve Geriatric Hip Fracture Outcomes: An Analysis of the ACS NSQIP Targeted Hip Fracture Series. J Orthop Trauma. 2019;33(6):e223–e8. Epub 2019/02/01. doi: 10.1097/BOT.0000000000001443 . 30702503

[47] Friedman SM, Mendelson DA, Bingham KW, Kates SL. Impact of a comanaged Geriatric Fracture Center on short-term hip fracture outcomes. Arch Intern Med. 2009;169(18):1712–7. Epub 2009/10/14. doi: 10.1001/archinternmed.2009.321 . 19822829

[48] Liu Y, Liu JP, Xia Y. Chinese herbal medicines for treating osteoporosis. Cochrane Database Syst Rev. 2014;(3):CD005467. Epub 2014/03/07. doi: 10.1002/14651858.CD005467.pub2 . 24599707 PMC10638660

[49] Shea B, Wells G, Cranney A, Zytaruk N, Robinson V, Griffith L, et al. Calcium supplementation on bone loss in postmenopausal women. Cochrane Database Syst Rev. 2004;(1):CD004526. Epub 2004/02/20. doi: 10.1002/14651858.CD004526.pub2 . 14974070

[50] Klop C, Gibson-Smith D, Elders PJ, Welsing PM, Leufkens HG, Harvey NC, et al. Anti-osteoporosis drug prescribing after hip fracture in the UK: 2000–2010. Osteoporos Int. 2015;26(7):1919–28. Epub 2015/05/13. doi: 10.1007/s00198-015-3098-x . 25963232 PMC4483189

[51] Yao P, Bennett D, Mafham M, Lin X, Chen Z, Armitage J, et al. Vitamin D and Calcium for the Prevention of Fracture: A Systematic Review and Meta-analysis. JAMA Netw Open. 2019;2(12):e1917789. Epub 2019/12/21. doi: 10.1001/jamanetworkopen.2019.17789 . 31860103 PMC6991219

[52] Gill CE, Mitchell PJ, Clark J, Cornish J, Fergusson P, Gilchrist N, et al. Experience of a systematic approach to care and prevention of fragility fractures in New Zealand. Archives of Osteoporosis. 2022;17(1):108. doi: 10.1007/s11657-022-01138-1 35917039 PMC9344235

[53] Holland A, Moffat T. Gendered perceptions of osteoporosis: implications for youth prevention programs. Glob Health Promot. 2020;27(2):91–9. Epub 2019/04/30. doi: 10.1177/1757975918816705 . 31033426

[54] Shanthi Johnson C, McLeod W, Kennedy L, McLeod K. Osteoporosis health beliefs among younger and older men and women. Health Educ Behav. 2008;35(5):721–33. Epub 2007/07/03. doi: 10.1177/1090198107301331 . 17602100

[55] Minns Lowe CJ, Toye F, Barker KL. Men’s experiences of having osteoporosis vertebral fractures: a qualitative study using interpretative phenomenological analyses. Osteoporos Int. 2019;30(7):1403–12. Epub 2019/05/02. doi: 10.1007/s00198-019-04973-0 . 31041474

[56] Sale JEM, Yang A, Elliot-Gibson V, Jain R, Sujic R, Linton D, et al. Patients 80 + have similar medication initiation rates to those aged 50–79 in Ontario FLS. Osteoporos Int. 2021. Epub 2021/01/21. doi: 10.1007/s00198-020-05796-0 . 33471148

[57] Cauley JA. Defining ethnic and racial differences in osteoporosis and fragility fractures. Clinical orthopaedics and related research. 2011;469(7):1891–9. Epub 2011/03/25. doi: 10.1007/s11999-011-1863-5 . 21431462 PMC3111798

[58] Wright NC, Chen L, Saag KG, Brown CJ, Shikany JM, Curtis JR. Racial Disparities Exist in Outcomes After Major Fragility Fractures. J Am Geriatr Soc. 2020;68(8):1803–10. Epub 2020/04/28. doi: 10.1111/jgs.16455 . 32337717 PMC7935465

[59] Mikuls TR, Saag KG, George V, Mudano AS, Banerjee S. Racial disparities in the receipt of osteoporosis related healthcare among community-dwelling older women with arthritis and previous fracture. J Rheumatol. 2005;32(5):870–5. Epub 2005/05/04. . 15868624

[60] Curtis JR, McClure LA, Delzell E, Howard VJ, Orwoll E, Saag KG, et al. Population-based fracture risk assessment and osteoporosis treatment disparities by race and gender. J Gen Intern Med. 2009;24(8):956–62. Epub 2009/06/25. doi: 10.1007/s11606-009-1031-8 . 19551449 PMC2710475

[61] Leslie WD, Brennan SL, Prior HJ, Lix LM, Metge C, Elias B. The post-fracture care gap among Canadian First Nations peoples: a retrospective cohort study. Osteoporos Int. 2012;23(3):929–36. Epub 2012/01/04. doi: 10.1007/s00198-011-1880-y . 22212736

[62] Leslie WD, Derksen S, Metge C, Lix LM, Salamon EA, Wood Steiman P, et al. Fracture risk among First Nations people: a retrospective matched cohort study. Cmaj. 2004;171(8):869–73. Epub 2004/10/13. doi: 10.1503/cmaj.1031624 . 15477625 PMC522652

[63] Amir O, Berry SD, Zullo AR, Kiel DP, Zhang T. Incidence of hip fracture in Native American residents of U.S. nursing homes. Bone. 2019;123:204–10. Epub 2019/04/06. doi: 10.1016/j.bone.2019.04.001 . 30951886 PMC6527125

[64] Pratt WB, Holloway JM. Incidence of hip fracture in Alaska Inuit people: 1979–89 and 1996–99. Alaska Med. 2001;43(1):2–5. Epub 2001/05/11. . 11345855

[65] Dy CJ, Lamont LE, Ton QV, Lane JM. Sex and gender considerations in male patients with osteoporosis. Clinical orthopaedics and related research. 2011;469(7):1906–12. Epub 2011/03/15. doi: 10.1007/s11999-011-1849-3 . 21400003 PMC3111783

[66] Johnson JL, Greaves L, Repta R. Better science with sex and gender: Facilitating the use of a sex and gender-based analysis in health research. Int J Equity Health. 2009;8:14. Epub 2009/05/08. doi: 10.1186/1475-9276-8-14 . 19419579 PMC2689237

[67] Allen VC Jr., Lachance C, Rios-Ellis B, Kaphingst KA. Issues in the Assessment of "Race" among Latinos: Implications for Research and Policy. Hisp J Behav Sci. 2011;33(4):411–24. Epub 2012/12/15. doi: 10.1177/0739986311422880 . 23239903 PMC3519364

[68] Kaphingst KA, Lachance CR, Gepp A, D’Anna LH, Rios-Ellis B. Educating underserved Latino communities about family health history using lay health advisors. Public Health Genomics. 2011;14(4–5):211–21. Epub 2010/01/07. doi: 10.1159/000272456 . 20051669 PMC3136386

[69] Itzigsohn J, Dore-Cabral C, editors. Competing identities? Race, ethnicity and panethnicity among Dominicans in the United States. Sociological Forum; 2000: Springer.

[70] Golash-Boza T, Darity W Jr. Latino racial choices: the effects of skin colour and discrimination on Latinos’ and Latinas’ racial self-identifications. Ethnic and Racial Studies. 2008;31(5):899–934.

[71] Vaquera E, Kao G. The implications of choosing “no race” on the salience of Hispanic identity: How racial and ethnic backgrounds intersect among Hispanic adolescents. The Sociological Quarterly. 2006;47(3):375–96.

[72] Landale NS, Oropesa RS. White, black, or Puerto Rican? Racial self-identification among mainland and island Puerto Ricans. Social Forces. 2002;81(1):231–54.

[73] Holley LC, Salas LM, Marsiglia FF, Yabiku ST, Fitzharris B, Jackson KF. Youths of Mexican Descent of the Southwest: Exploring Differences in Ethnic Labels. Child Sch. 2009;31(1):15–26. Epub 2009/10/10. doi: 10.1093/cs/31.1.15 . 19816593 PMC2758796

