# Supplementary material for: Association of age, sex and race with prescription of anti-osteoporosis medications following low-energy hip fracture in a retrospective registry cohort
Source: PLoS One. 2022 Dec 1;17(12):e0278368. doi: 10.1371/journal.pone.0278368 (PMC9714945; doi:10.1371/journal.pone.0278368)
Supplement: S1 Table — (DOCX) [file pone.0278368.s001.docx]

**S1 Table. Common Procedural Terminology (CPT) codes used for cohort building**

| **CPT Code** | **Description** |
| --- | --- |
| **27236** | Open treatment of femoral fracture, proximal end, neck, internal fixation or prosthetic replacement |
| **27244** | Treatment of intertrochanteric, pertrochanteric, or subtrochanteric femoral fracture; with plate/screw type implant, with or without cerclage |
| **27245** | Open treatment of intertrochanteric, pertrochanteric, or subtrochanteric femoral fracture, with intramedullary implant, with or without interlocking screws and/or cerclage |
