# Supplementary material for: Association of age, sex and race with prescription of anti-osteoporosis medications following low-energy hip fracture in a retrospective registry cohort
Source: PLoS One. 2022 Dec 1;17(12):e0278368. doi: 10.1371/journal.pone.0278368 (PMC9714945; doi:10.1371/journal.pone.0278368)
Supplement: S2 Table — (DOCX) [file pone.0278368.s002.docx]

**S2 Table. Adjusted Associations of Exposures and Covariates with Prescription of Anti-Osteoporosis Medication inclusive of All Covariates**

|  | **Odds Ratio^a^**  **(95% CI)** | **P Value^a^** |
| --- | --- | --- |
| **Main Exposures** | | |
| Age (Decade) | - | 0.14 |
| Male sex | - | 0.01 |
| Age* Sex | - | 0.029 |
| Male sex within patients age 50-59 | 0.75 (0.60-0.92) | 0.007 |
| Male sex within patients age 60-69 | 0.81 (0.70-0.94) | 0.005 |
| Male sex within patients age 70-79 | 0.89 (0.81-0.97) | 0.011 |
| Male sex within patients age 80-89 | 0.96 (0.87-1.07) | 0.47 |
| Male sex within patients age 90+ | 1.05 (0.90-1.23) | 0.55 |
| Age within patients of female sex | 0.96 (0.92-1.01) | 0.14 |
| Age within patients of male sex | 1.05 (0.98-1.12) | 0.15 |
| Race (Reference: White) | - | 0.036 |
| Asian | 0.83 (0.62-1.11) | 0.21 |
| Black | 0.87 (0.70-1.08) | 0.21 |
| Hispanic | 0.94 (0.76-1.16) | 0.58 |
| Indigenous | 2.01 (1.19-3.38) | 0.009 |
| **Included Covariates** |  |  |
| BMI | 0.99 (0.989-0.999) | 0.031 |
| ASA Classification | 1.04 (0.97-1.12) | 0.27 |
| MFI5 Score | 0.93 (0.89-0.98) | 0.009 |
| Medical Co-Management (Reference=Yes) | 1.01 (0.83-1.23) | 0.92 |
| Standardized Hip Fracture Program (Reference: Yes) | 0.61 (0.57-0.67) | <0.001 |
| Fixation (Reference: Replacement) | 0.91 (0.84-0.99) | 0.029 |
| Total Length of Stay, days | 0.99 (0.98-0.999) | 0.028 |

Abbreviations: CI: confidence interval.

^a^Odds ratios, 95% CIs, and p-values produced using multivariable logistic regression.
