# Supplementary material for: Association of age, sex and race with prescription of anti-osteoporosis medications following low-energy hip fracture in a retrospective registry cohort
Source: PLoS One. 2022 Dec 1;17(12):e0278368. doi: 10.1371/journal.pone.0278368 (PMC9714945; doi:10.1371/journal.pone.0278368)
Supplement: S3 Table — (DOCX) [file pone.0278368.s003.docx]

**S3 Table: Sensitivity Analysis: Adjusted Associations of Exposures and Covariates with Prescription of Anti-Osteoporosis Medication Inclusive of those with unknown race**

| **Total n=16,657** | **Odds Ratio^a^**  **(95% CI)** | **P Value^a^** |
| --- | --- | --- |
| **Main Exposures** | | |
| Age (Decade) | - | 0.65 |
| Male sex | - | 0.002 |
| Age* Sex | - | 0.029 |
| Male sex within patients age 50-59 | 0.74 (0.61-0.88) | <0.001 |
| Male sex within patients age 60-69 | 0.80 (0.70-0.90) | <0.001 |
| Male sex within patients age 70-79 | 0.86 (0.79-0.93) | <0.001 |
| Male sex within patients age 80-89 | 0.93 (0.85-1.00) | 0.06 |
| Male sex within patients age 90+ | 1.00 (0.88-1.13) | 0.97 |
| Age within patients of female sex | 1.01 (0.97-1.05) | 0.65 |
| Age within patients of male sex | 1.09 (1.03-1.15) | 0.003 |
| Race (Reference: White) | - | 0.036 |
| Asian | 0.83 (0.62-1.11) | 0.21 |
| Black | 0.87 (0.70-1.08) | 0.21 |
| Hispanic | 0.94 (0.76-1.16) | 0.58 |
| Indigenous | 2.15 (1.28-3.63) | 0.009 |
| Unknown | 2.32 (2.12-2.54) | <0.001 |

Abbreviations: CI: confidence interval.

^a^Odds ratios, 95% CIs, and p-values produced using multivariable logistic regression adjusting for BMI, ASA class, mFI5 comorbidity score, fracture location, type of surgical treatment, hospital length of stay, whether or not they were treated at a hospital with a standardized hip fracture program and whether or not they were co-managed by a medical specialty physician.
